# Diverse origin of *Plasmodium falciparum* in northwest Ecuador

**DOI:** 10.1186/s12936-019-2891-y

**Published:** 2019-07-26

**Authors:** Claudia A. Vera-Arias, L. Enrique Castro, Javier Gómez-Obando, Fabián E. Sáenz

**Affiliations:** 1 Centro de Investigación para la Salud en América Latina, Facultad de Ciencias Exactas y Naturales, Pontificia Universidad Católica del Ecuador, Av. 12 de octubre 1076, Apartado: 17-01-2184, Quito, Ecuador; 2 Ministerio de Salud Pública, Guayaquil, Ecuador; 3 Ministerio de Salud Pública, Distrito de Salud de San Lorenzo, San Lorenzo, Ecuador

**Keywords:** Malaria, *Plasmodium falciparum*, Microsatellites, Genetic structure, Ecuador

## Abstract

**Background:**

Ecuador plans to eliminate malaria by 2020, and the country has already seen a decrease in the number of cases from more than 100,000 in 2000 to only 618 in 2015. Around 30% of malaria infections in Ecuador are caused by *Plasmodium falciparum*. Most malaria population genetics studies performed in Latin America, especially in the Pacific Coast, indicate a high clonality and a clear structure of *P. falciparum* populations. It was shown that an outbreak of *P. falciparum* in northwest Ecuador was the result of a clonal expansion of parasites circulating at low levels in the country or re-invading Ecuador from neighbouring territories. However, general characteristics of *P. falciparum* circulating in the northwest coast of Ecuador have not been determined. The main goal of this study was to genetically characterize the population structure of *P. falciparum* in coastal Ecuadorian localities bordering with Colombia.

**Methods:**

Molecular investigation of 41 samples collected from 2013 to 2016 in San Lorenzo County, northwest Ecuador was performed using seven neutral microsatellite markers.

**Results:**

The genetic population structure of *P. falciparum* in northwest Ecuador is clearly defined as three different genetic groups previously reported in Ecuador, Peru and Colombia.

**Conclusions:**

The limited number of *P. falciparum* clonal types that are circulating in northwest Ecuador, are related to ancestral parasite clonal lineages reported in the Pacific Coast. These parasites could be a product of migration from neighbouring regions or residual clonal types circulating in the country in low proportions. Studies of the genetic characterization of *P. falciparum* in eliminating areas help determine the possible origin of parasites in order to create strategies to prevent the entrance of new lineages and achieve local elimination of malaria.

## Background

Malaria is present in 21 countries of Latin America, and about 126.8 million people were at risk of the disease in 2016 in the region. *Plasmodium falciparum* was responsible for approximately 30% of the reported malaria cases in the region [1–5]. Ecuador is one of the eight countries of the region with capacity to eliminate malaria by 2020 [1]; indeed, the country has decreased the number of cases from more than 100,000 in 2000 to 618 in 2015, and 1279 in 2017 [1, 4, 6]. In Ecuador, the presence of malaria is mostly restricted to the northwest coast and the Amazon region, where outbreaks of *P. falciparum* and *Plasmodium vivax* still occur [6, 7].

Genetic characterization of circulating malaria parasites in a specific area, especially in areas targeted for elimination, provide insights about the genetic connectivity of currently circulating populations to ancestral lineages and determine if left over residual historical parasite lineages are contributing to local transmission. This will also help to determine if new parasite lineages that have migrated from other regions are contributing to current malaria transmission. In addition, it can provide data about drug resistant alleles that may be relevant for targeting appropriate drugs for treatment or for mass drug administration [8–10]. Moreover, the level of diversity and its distribution provide insights into trends in parasite transmission and population history [11, 12].

Most malaria population genetics studies performed in Latin America indicate high clonality of *P. falciparum* populations [13–16]. *Plasmodium falciparum* from Ecuador, Colombia, Peru, Honduras, Brazil and Venezuela have undergone one or more bottleneck events in the recent past and current populations expanded from a limited number of *P. falciparum* ancestral lineages [7, 17–20]. *Plasmodium falciparum* populations in the region consist of a continuous mixture and reorganization of clonal lineages (genetically identical for a set of markers, but potentially variable for others [9]), mainly due to migration, even though the opportunities for outcrossing between the different lineages is limited because of low transmission [9, 13, 17, 21]. In addition, *P. falciparum* from Latin America has had chloroquine (CQ) resistance since 1960 [20], as well as sulfadoxine–pyrimethamine (SP) resistance [22, 23]. Recently, artemisinin (ART) resistance-related mutations have been reported in Guyana [20, 24, 25].

A molecular investigation of Peruvian *P. falciparum* population determined the presence of five clonal lineages in the country in 1999–2000. The Peruvian *P. falciparum* population consisted of A, B, C, D and E lineages distributed across the country. In the Amazon interior region, the five clonal lineages were present and in the northern Pacific coast only one lineage was reported (clonal lineage E). Each clonet had a specific drug resistant allelic profile; while all clonets reported CQ resistance, the clonets D and E had *dhfr* and *dhps* alleles that confer SP sensitivity [21]. A *P. falciparum* outbreak in Tumbes (Pacific coast of Peru) during 2010–2012, had a genotype related to clonal lineage B (B_v1_) but was unrelated to clonal lineage E (previously present in the same area) [15] and suggested that this outbreak was caused by clonal lineages from the Amazon region of Peru. Similarly, in Colombia the *P. falciparum* population has undergone a bottleneck event, showing low genetic diversity and low polyclonal infections [26]. The *P. falciparum* population structure consisted of four major clusters along the Colombian Pacific coast between 1999 and 2009 [26]. A different study in Colombia showed several multilocus haplotypes persist in multiple years between 2003 and 2010 in most of the country in Amazonas, Cordoba, Nariño and Valle [27]. Clonal lineage B_v1_ (reported in Peru [14]) in the Amazon, two new clusters F in Nariño, Valle and Cauca and cluster E_V1_ in Antioquia were reported [14, 16, 26–28]. Colombian parasites have reported CQ and SP resistance, in addition to an increase in the number of *pfmdr*-*1* copies, carrying mefloquine (MQ) and quinine (QN) resistance. Neither Peru nor Colombia has reported ART resistance or mutations in the Kelch 13 propeller domain [20, 22–25, 29].

The information about *P. falciparum* population genetic structure in Ecuador is limited. A molecular study of *P. falciparum* from Ecuador, during an outbreak in Esmeraldas city in the northwest of the country between November 2012 and November 2013, revealed that the parasites were the result of a clonal expansion of *P. falciparum* circulating at low levels or re-invading Ecuador from border countries [7]. The *P. falciparum* outbreak in northwest Ecuador had an identical microsatellite genotypic profile to *P. falciparum* E clonal lineages from the Peruvian Pacific coast. Interestingly, these parasites were related to a single historical isolate that was collected in the Ecuadorian coast in 1990 [7]. Esmeraldas outbreak samples carried CQ resistance (CVMNT haplotype) and *dhfr* and *dhps* alleles that were similar to E clonal type that were associated with SP sensitivity [7].

Molecular tools like neutral microsatellite markers (tandem repeats of motifs [18]) are a very important and powerful tool for the study of population structure because they can characterize and identify haplotypes and are extremely widespread in *P. falciparum* (2–3 kb throughout the genome) [12, 18, 30]. Neutral microsatellites are usually the markers of choice for *P. falciparum* population genetic analysis because these markers are not directly under selection and are able to show genetic signatures [13–16, 18, 30]. There is considerable amount of data using neutral microsatellite markers that have provided clues about genetic connectivity between parasite populations in Peru, Ecuador and Colombia.

The main goal of this study was to genetically characterize and geographically map the population structure of *P. falciparum* in northwest Ecuador (San Lorenzo county), between 2013 and 2016 using seven neutral microsatellites markers and compare them to previously characterized Ecuadorian parasites. In addition, Ecuadorian *P. falciparum* genotypes were compared to Peruvian and Colombian parasites.

## Methods

### Ethics statement

The parasite samples used in this study were obtained from the malaria surveillance program from the Ecuadorian Ministry of Health. The protocol was approved by the Ethical Review Committee of Pontificia Universidad Católica del Ecuador (approvals #: CBE-016-2013 and 20-11-14-01). Written informed consent was provided by study participants and/or their legal guardians.

### Study sites

**Fig. 1:**
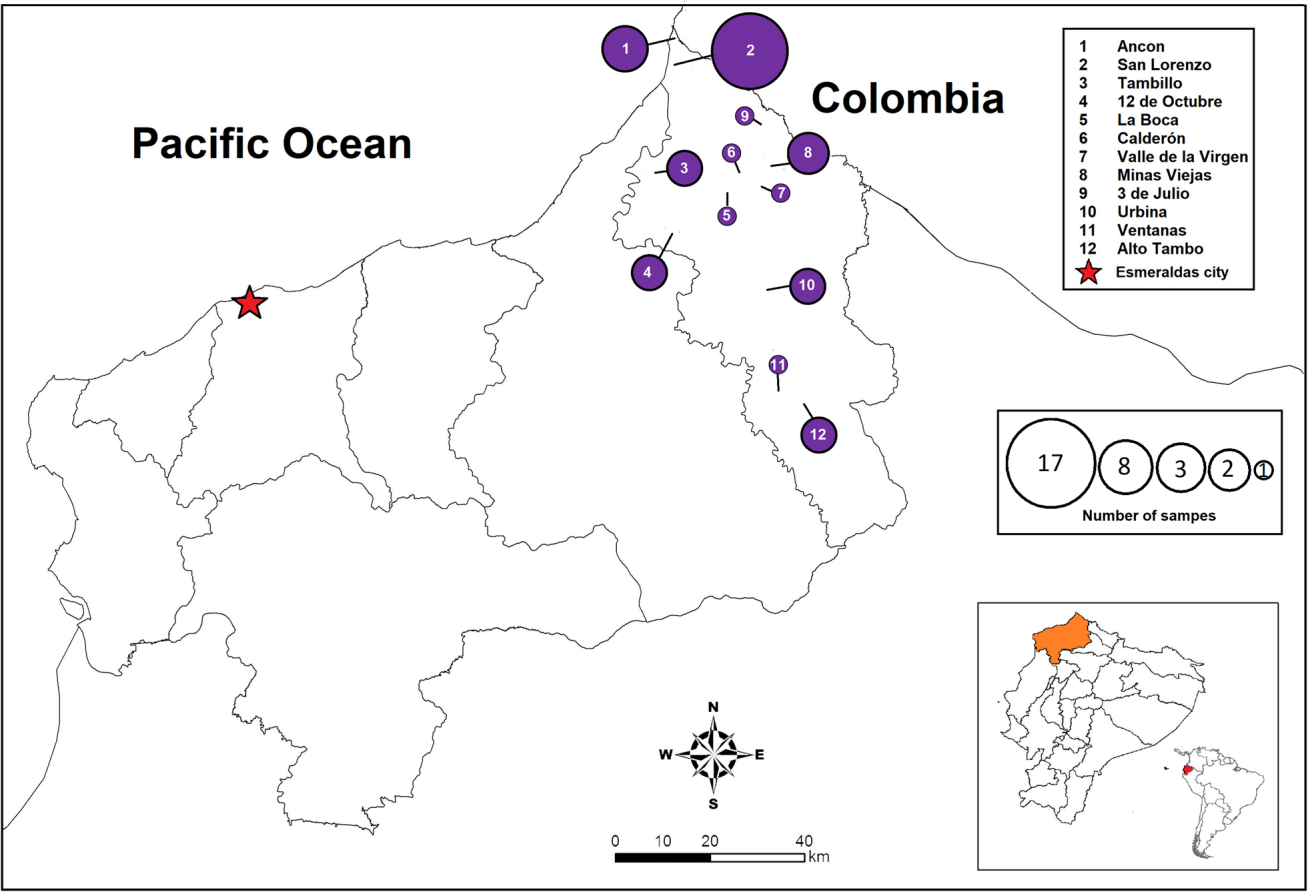
Sample number and collection sites. All samples were collected in Esmeraldas province, San Lorenzo county, northwest Ecuador: there were 12 collection communities. Each sample collection location is represented with a purple circle. The circle diameter is proportional to the number of samples collected in each location

The study analysed samples from communities in San Lorenzo county, northwest Ecuador (Fig. 1). These samples were compared to previously reported samples from the same province [7]. All the samples used in this study were collected between 2013 and 2016 (Fig. 1). Ecuadorian parasites were compared to previously reported Peruvian and Colombian parasites [21, 26].

### Samples and reference isolates

Samples used in this study were collected from patients between January 2013 and March 2016 by the personnel of the Ministry of Health of Ecuador. A total of 41 blood samples (4 samples in 2013; 15 in 2014; 14 in 2015 and 8 in 2016) were collected in San Lorenzo county from patients who initially reported to be microscopically positive for *P. falciparum* infection, and from whom informed consent was obtained. The blood samples were collected by finger prick or by drawing peripheral whole blood and spotted on 3MM Whatman filter paper. Thirty-two samples from an outbreak in Esmeraldas city, between November 2012 and November 2013 and reported in Saenz et al. [7] were also included in the study. Fifty-six percent of the samples (23/41) were collected by finger prick or by drawing peripheral whole blood and 44% of samples (18/41) were collected only by rapid diagnostic tests (RDT) with a positive result.

### DNA extraction and confirmation of infection

DNA was isolated from all the samples (whole blood, filter paper or RDT) using a QIAamp DNA MINI KIT (QIAGEN Sample & Assay Technologies, Germantown, USA). *Plasmodium falciparum* was confirmed by two different molecular methods: nested PCR using primers for 18S ribosomal [31] and PET-PCR using photo-induced electron transfer fluorogenic primers [32].

### Microsatellite typing

Genomic DNA was used for microsatellite characterization. Samples were genotyped for seven neutral microsatellite loci spanning six chromosomes (TA1, chromosome 6; Polyα, ch. 4; PfPk2, ch. 12; TA109, ch. 6; 2490, ch. 10; C2M34, ch. 2; C3M69, ch. 3) [12, 18, 33]. DNA was amplified using PCR previously described methods [12, 18, 21, 34, 35]. PCR products were labelled with fluorescent dyes (FAM or HEX) and were separated by capillary electrophoresis on an Applied Biosystems 3130xl genetic analyzer in South Korea at Macrogen Company. The fragments were scored using Peak Scanner Software V1.0 (Applied Biosystems Foster City, USA) [36].

### Statistical analysis

Heterozygosity (He) was estimated using the formula $He = \frac{n}{n - 1} \left( {1 - \mathop \sum \nolimits_{i = l}^{k} p_{1}^{2} } \right)$, and pairwise fixation indices (Fst), estimated using the formula $\left( {\delta \mu } \right)^{2} = \left( {\mu_{A} - \mu_{B} } \right)^{2}$, where $\left( {\delta \mu } \right)^{2}$ is the genetic distance and $\mu_{A}$ and $\mu_{B}$ are the average number of allelic size differences within population A and B, respectively. He and Fst were calculated using Arlequin 3.5.1.2 software (CMPG, Swiss Institute of Bio-informatics, Lausanne, Switzerland) [37, 38]. To assess the parasite population structure, the genetic data was analysed with Structure 2.3.4 software, that assigned samples to different populations (K), and Structure Harvester was used to define the number of expected populations [39–41]. The data were evaluated using different K values (K = 1–15) and twenty independent repetitions were run for each K value with a burn-in period of 10,000 iterations followed by 1,000,000 iterations. In addition, software PHYLOViZ version 10 was used to construct a median-joining network diagram [42]. The samples were represented in a complete spanning tree using goeBURST algorithm. This analysis shows the possible evolutionary relationships between strains (samples) [42].

## Results

### Genetic characterization of Ecuadorian *Plasmodium falciparum* using neutral microsatellite markers

To determine the genetic composition of isolates from northwest Ecuador, Esmeraldas province, San Lorenzo county, between 2013 and 2016, seven neutral microsatellite markers located in six different chromosomes were genotyped. The markers showed the presence of different alleles. The marker TA1 had two alleles: the allele 171, present in 90% of the samples (37/41), and the allele 174, present in 7% of the samples (3/41). The marker Poly-α was one of the most diverse with five alleles: the allele 180 was the most common (42%) in all the samples, while 34% of the samples (14/41) had the allele 147, the allele 183 was present in 15% of the samples (6/41) and the allele 174 was present in 7% (3/41) of the samples, the allele 177 was present in 2% (1/41) of the samples. PfPK2 also had five alleles, being the most common 168 in 44% of the samples (18/41), 39% (16/41) of the samples had the allele 174, 7% (3/41) of the samples had the alleles 171 and 159 and 2% (1/41) had the allele 177. The marker TA109 was the least diverse marker, with the allele 160 present in all the samples. The microsatellite 2490 showed three alleles, being the most common 81 in 56% (23/41) of the samples, 72 in 22% (9/41) of the samples and 78 in 15% (6/41) of the samples. The marker C2M34 had five alleles and the most common was 224 in 51% of samples (21/41), 29% (12/41) had the allele 226, the alleles 222 and 232 were present in 5% (2/41) of the samples and the less common allele was 230 in 2% (1/41) of the samples. The marker C3M69 had two alleles, 90% of samples (37/41) had the allele 140 and 7% (3/41) of samples had the allele 122.

San Lorenzo samples showed a higher heterozygosity (He = 0.4726) than Esmeraldas (He = 0.1018) [7] (Table 1) and Linkage Disequilibrium (LD) in San Lorenzo (I_A_^S^ = 0.0762, p < 1.00 × 10^−2^) was much lower than in Esmeraldas (I_A_^S^ = 0.3798, p < 1.00 × 10^−2^) [7] (Table 1). Pairwise Fst between the two Ecuadorian counties (Esmeraldas and San Lorenzo) was high (0.39640) [43] (Table 2).

**Table 1:** Number of alleles, heterozygosity (He) and linkage disequilibrium (LD) of *P. falciparum* in northwest Ecuador

Groups	Number of samples	Number of alleles	He	LD (I_A_^S^)
San Lorenzo	41	3.6670	0.4726	0.0762
Esmeraldas	34	2.4000	0.1018	0.3798

**Table 2:** Pairwise Fst of Ecuadorian, Peruvian [21] and Colombian samples [26]

Location	No. samples	Esmeraldas	San Lorenzo
Esmeraldas (ECU)	34	0.00000	
San Lorenzo (ECU)	41	0.39640	0.00000
Zarumilla (PER)	42	0.15103	0.29417
West Amazon (PER)	15	0.77968	0.35719
Nariño (COL)	34	0.45433	0.08481
Valle (COL)	25	0.46825	0.06966

**Fig. 2:**
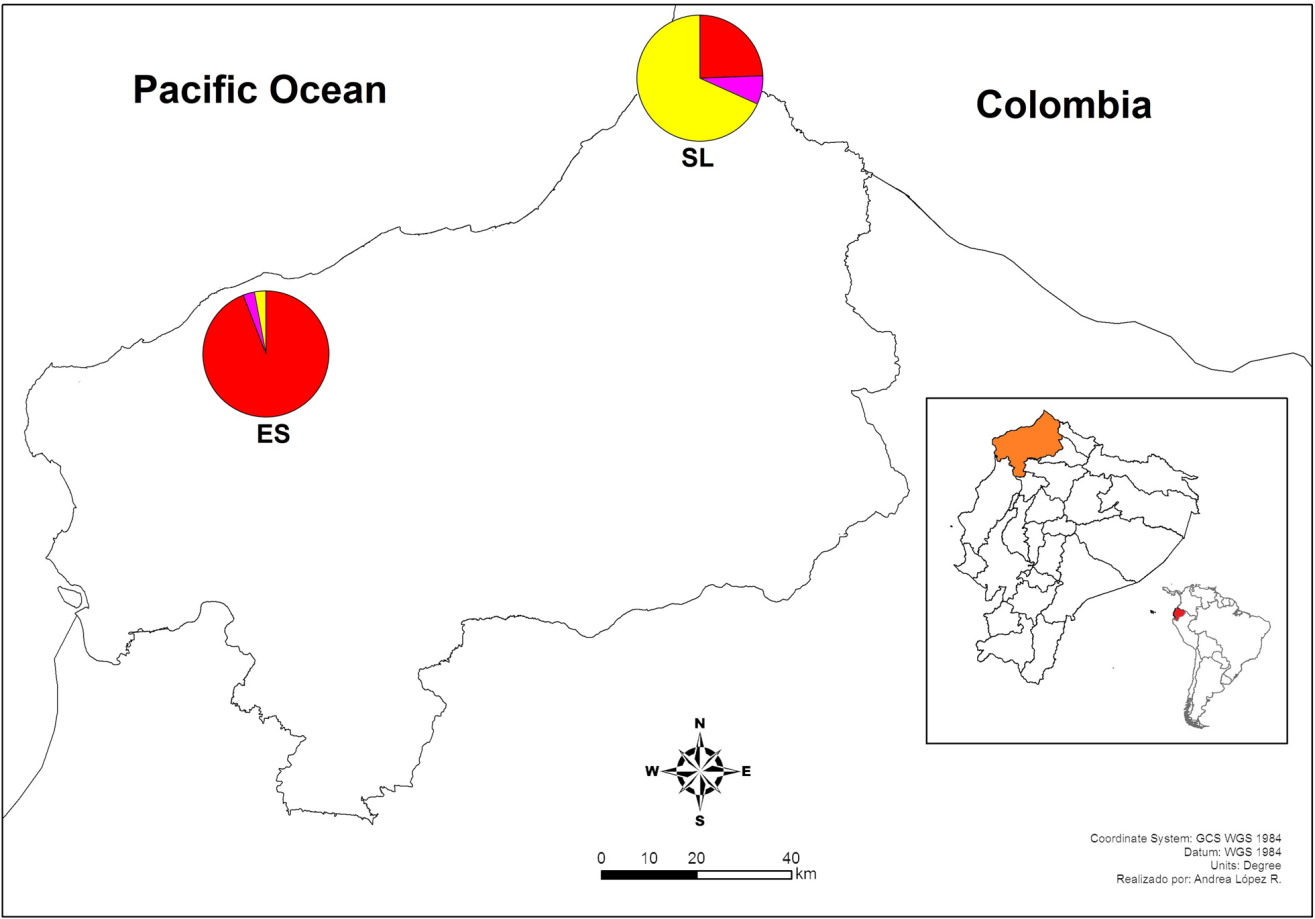
Population structure of *Plasmodium falciparum* samples from northwest Ecuador (N = 75). SL: San Lorenzo County, ES: Esmeraldas County. Distribution of the three clusters (red, pink and yellow) across northwest Ecuador. San Lorenzo County had three clusters. Red—Cluster 1 (N = 10); Yellow—Cluster 3 (N = 28); Pink—Cluster 4 (N = 3). Esmeraldas County had three clusters. Red—Cluster 1 (N = 32); Yellow—Cluster 3 (N = 1); Pink—Cluster 4 (N = 1)

Cluster analysis inferred a well differentiated population structure in this group of isolates. Three population clusters (K) were predicted in northwest Ecuador (San Lorenzo and Esmeraldas) (Fig. 2). Most samples from San Lorenzo county 68.3% (28/41) belonged to cluster one (Yellow); 24.4% (10/41) belonged to cluster two (Red) and 7.3% (3/41) belonged to cluster three (Pink) (Fig. 2). This information was confirmed by a Neighbour Joining network. Thirteen different haplotypes formed the yellow cluster, three haplotypes formed the red cluster, and two haplotypes were part of the pink cluster.

### Genetic relatedness between Ecuadorian, Peruvian and Colombian isolates

The comparison between Ecuadorian (2013–2016), Peruvian (1999–2012) [21] and Colombian (2003–2008) samples [26] showed close relationship between samples of the three countries. Pairwise Fst was performed between samples from northwest Ecuador and samples from neighboring locations of Peru and Colombia. Ecuadorian samples from San Lorenzo had a close genetic relationship with Valle (0.06966) and Nariño (0.08481) but were distantly related from the Peruvian West Amazon and the Pacific Coast of Peru (Table 2).

**Fig. 3:**
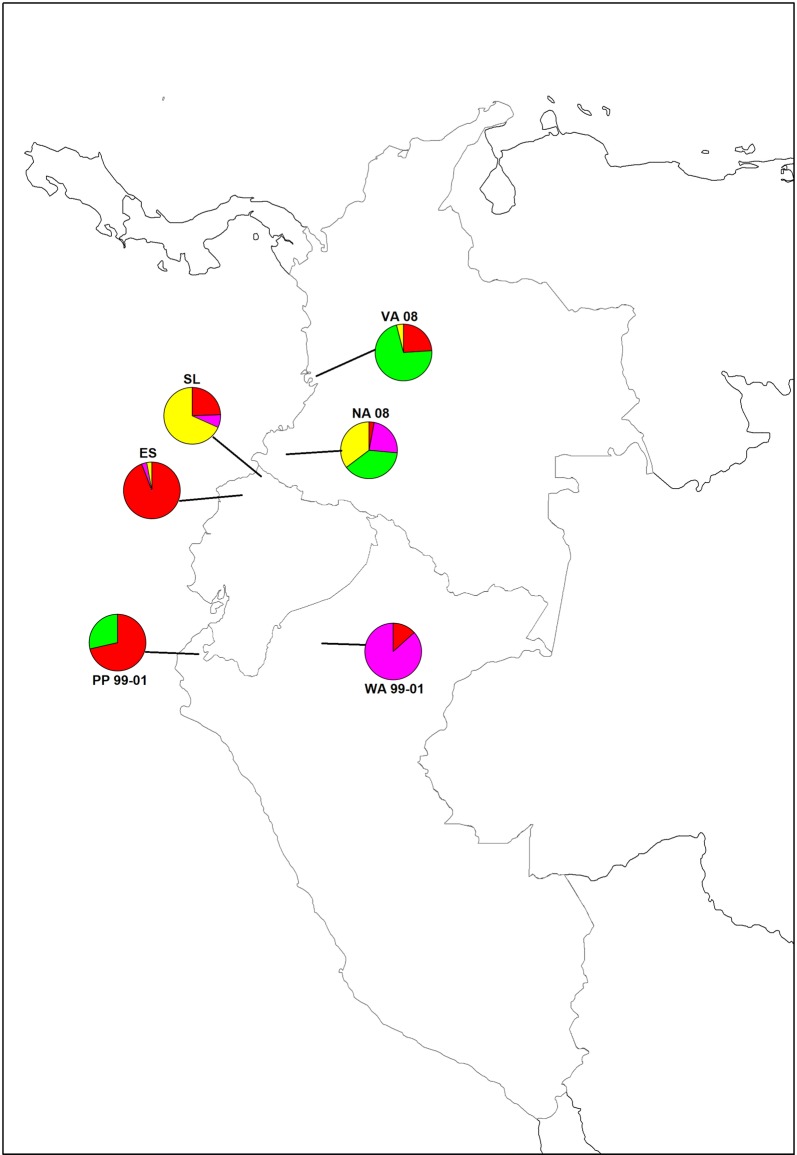
Population structure of *Plasmodium falciparum* samples from Ecuador (N = 75), Colombia (N = 59) [26] and Peru (N = 57) [21]. SL: San Lorenzo County, ES: Esmeraldas County, VA: Valle, NA: Nariño, PP: Zarumilla, WA: West Amazon from Peru. Distribution of the four clusters across Ecuador, Colombia and Peru. Red—Cluster 1 (N = 81); Green—Cluster 2 (N = 43); Yellow—Cluster 3 (N = 42); Pink—Cluster 4 (N = 25)

The comparison of localities using Structure software predicted the presence of four clusters in San Lorenzo, northwest Ecuador and its neighbouring locations. All samples (41/41) from San Lorenzo corresponded to three clusters (yellow, red and pink). The yellow cluster was predominantly present in Nariño [26], Colombia (35%), the Red cluster was the most prevalent parasite in Esmeraldas (94%), the pacific Coast of Peru [21] (71%) and was present in Valle [26] (24%) and the West Amazon of Peru [21] (13%) (Fig. 3), while the pink cluster was predominant in Peru’s West Amazon [21] (87%), it was present in Nariño [26], Colombia (24%), and in Esmeraldas [7] (3%).

**Fig. 4:**
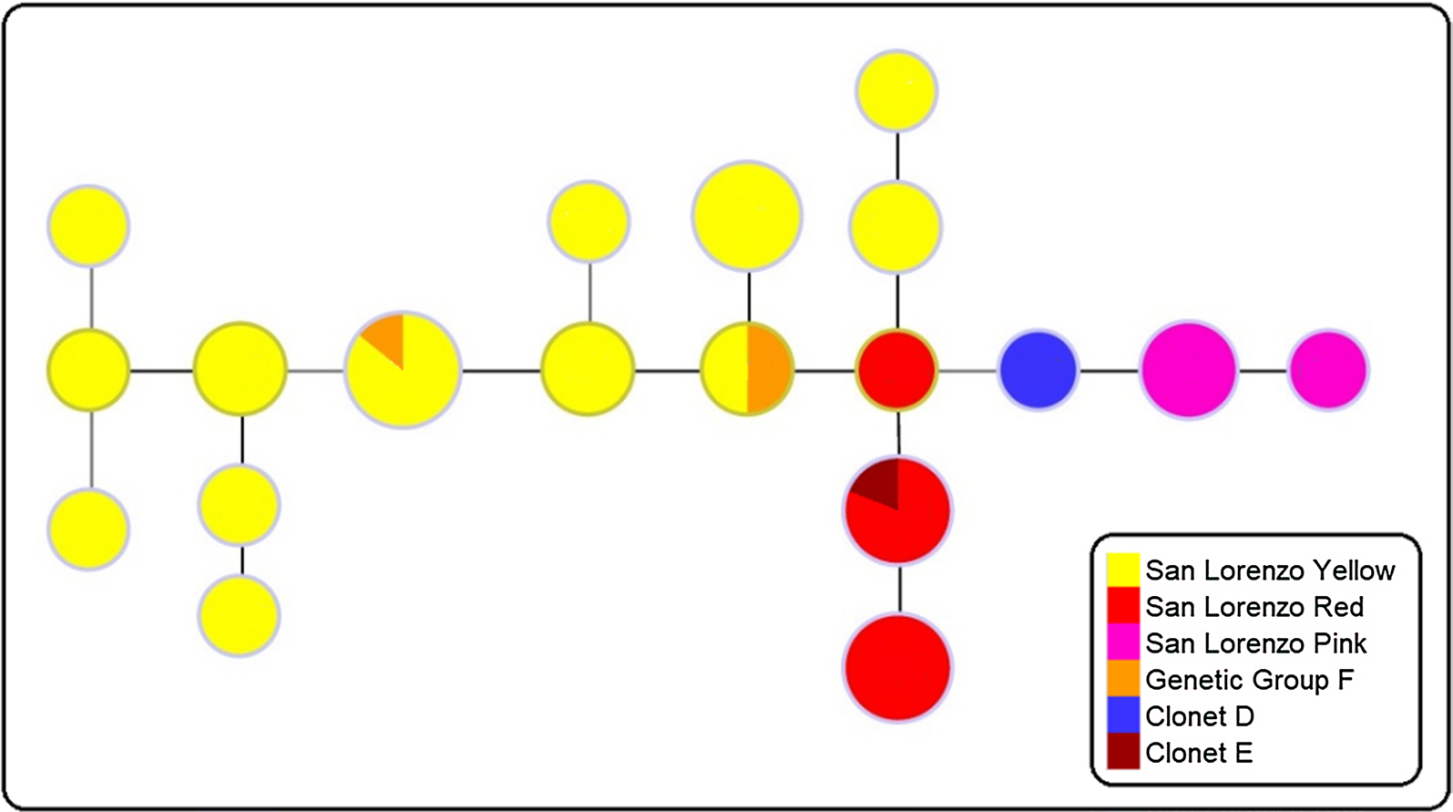
Median-joining network diagram of Ecuadorian *P. falciparum* and previously reported clonets. The network diagram shows the genetic relationship between samples from San Lorenzo county, Esmeraldas county [7], Colombian genetic group F [27] and Peruvian clonets D and E [21]. The circle size is proportional to the number of samples with the same haplotype. The colors represent clusters assigned by Structure software

In order to identify if the parasites from northwest Ecuador corresponded to any of the previously reported clonal lineages or genetic clusters, a median-joining network was completed using predominant haplotypes of clonets D, E [21] and F [27]. The network diagram shows that *P. falciparum* from San Lorenzo’s Yellow cluster are closely related to genetic lineage F from Colombia (two haplotypes shared with previously reported haplotype F) [27]. In addition, parasites in the Red cluster are closely related to E clonal lineage parasites present in Esmeraldas and previously in the North coast of Peru [7, 21]. Finally, parasites in the Pink cluster were related to clonal lineage D, first described in the Amazon of Peru and reported in Esmeraldas [7, 21] (Fig. 4). The main haplotypes for each previously described lineage and the parasites in this study are shown in Table 3.

**Table 3:** Main microsatellite and drug resistance markers haplotypes [47] present in northwest Ecuador as compared to previously described haplotypes

	TA1	Poly-α	PfPK2	TA109	2490	C2M34	C3M69	Pfcrt	Pfdhfr	Pfdhps	Pfmdr1
San Lorenzo Yellow	171/174	147/177/180/183	159/168/171/174/177	160	78/81	222/224/226	140	CVMNT	CNCSI	SAKAA	NEDFSDFD
Clonet F (Dorado)	171	147/180/183	159/168/171	160	78/81	224/226	140	–	–	–	–
San Lorenzo Red	171	147	174	160	72/81	226/224	140	CVMNT/CVMET	CNCSI/CICNI	SAKAA	NEDFSDFD
Esmeraldas clonet E (Saenz)	171	147	174	160	72	226	140	CVMNT	CNCSI	SAKAA	NEDFSDFD
San Lorenzo Pink	171	174	174	160	81	230/232	122	CVMET	CNCNI	SAKAA	NEDFSDFD
Clonet D (Griffing)	171	174	174	160	81	232	122	CVMNT	CNCNI	SA (syn)AA	NEDFCDFD/NEDFCDFY

## Discussion

In Ecuador *Plasmodium* infections are reported in the Amazon and Costal regions [1, 7]. Specifically, the northwest coast of Ecuador has historically been endemic to *P. falciparum* where periodic transmission of this parasite at low levels has been reported [7]. This study was designed to understand the *P. falciparum* parasite population structure in parasite isolates collected in recent years and determine how this data can be used in support of malaria elimination efforts.

This study employed seven neutral microsatellites (TA1, Poly-α, PfPK2, TA109, 2490, C2M34 and C3M69 [12, 18, 33]) to characterize *P. falciparum* populations from Esmeraldas Province in northwest Ecuador. The same seven markers have been widely used in South America to characterize *P. falciparum* populations in Peru [15, 21], Colombia [26, 27] and Brazil [44].

*Plasmodium falciparum* from northwest Ecuador have medium/low diversity (medium/low He and medium/high linkage disequilibrium) similarly to what has been reported for other places of South America. This is partly because *P. falciparum* populations have undergone bottleneck events in the recent past due to elimination efforts by malaria programmes [14, 21, 26].

When comparing San Lorenzo (border locality) with Esmeraldas (150 km from border), it is clear that the border locality has more diversity and has different genetic composition from less endemic localities. This is due to two main factors: (1) regular migration from Colombia is common in the border areas and (2) most samples collected in Esmeraldas city were from a clonal *P. falciparum* outbreak [7]. Low LD in border localities matches higher number parasites entering from Colombia into Ecuador and a higher number of cases in the border county of San Lorenzo [45, 46].

This study shows that northwest Ecuador has a simple, well defined structure. Indeed, between 2013 and 2016, three different genetic groups were present. These groups are related to previously reported groups in Colombia, Peru and Ecuador itself.

The majority of samples (68.3%) had genetic similarity to samples circulating in Colombia. This cluster was previously defined as genetic lineage F by Dorado et al. [27]. The parasites in San Lorenzo, Ecuador shared the majority of markers with the defined F haplotype (Fig. 4 and Table 3) and only had some variations previously reported for F genetic lineage. This similarity is expected since the F clonet has been reported in the southern part of Colombia and human migration between Colombia and southern Ecuador is common. This human migration is related to several activities and some of the well-known include mining and palm oil agriculture. In addition, several Colombians and Ecuadorians cross the border on a daily basis for other reasons. This genetic lineage presents the drug resistance haplotype CVMNT and wild type *dhfr* and *dhps* drug resistance markers. The mutations 184S and 1042D in *Pfmdr1* are also related to genotypes reported previously [47].

One-fourth (28%) of the analysed samples from *P. falciparum* in San Lorenzo had genetic similarity or identity to parasites previously reported in an outbreak in Esmeraldas and the Pacific coast of Peru [7, 21]. This genetic type has also been reported in Valle, Colombia and in Nariño, Colombia with some variations. In addition, the E clonet was present in early 1990 in Esmeraldas province [7] suggesting that this group has been present in the area for several years. The parasites from clonet E have a characteristic conserved drug resistance genotype that includes a mutation in the 76 position of *Pfcrt* (CVMNT), prevalence of wild type genotypes for *Pfdhfr* and *Pfdhps* and mutations 184S and 1042D in *Pfmdr1* (Table 3) [7, 47].

A small percentage of the samples (7.3%) have similarity with D clonal type previously reported in the West Amazon of Peru [21], Esmeraldas [7] and Colombia [27]. The D clonet was first reported in the West Amazon of Peru in 1999–2000 but found in the South Pacific Coast of Colombia in 2008 and in the Ecuadorian coast in 2013 [7, 21, 26, 27]. The D clonet was found again in the north coast of Ecuador in this study in samples from 2013 and 2014 but not in more recent samples. As previously suggested by Griffing et al. [21], these clonal type parasites could have migrated to western Amazon of Peru from Ecuador which could have originated in Colombia and spread south to Ecuador [21]. The D clonet parasites in Ecuador have a characteristic *pfcrt* CVMET genotype and the synonymous mutation in the 540 position of *pfdhps* is common [7, 47].

In summary, this study showed that all the parasites that were found in the reported study sites clearly belonged to one of the three mentioned clusters that have been known to be present in the Pacific Coast of Peru, Ecuador and Colombia. It was difficult to determine if there are further variations between these clonal types found in Colombia, Ecuador and Peru using these limited genetic markers. However, future efforts can focus on characterizing the genotypes of these parasite types using genomic analysis. This data suggests that some ancestral populations that have been known to have existed in this region are still continuing to cause transmission of malaria in this region. Previous studies have also found that most of these parasites are carrying markers associated with CQ resistance but sensitive to SP. No evidence for artemisinin resistant genotypes were found. Collectively, these data suggest using current anti-malarial drug policies implemented in Ecuador these parasites can be treated during elimination phase. Continuous characterization of parasite isolates from this region using genomic analysis may help to determine if human migration between border regions of Ecuador and Colombia is a primary cause of malaria importation to Ecuador.

This study increases the knowledge about *P. falciparum* populations circulating in Ecuador and in the region. It gives a better understanding of the parasites present for future surveillance and prevention of parasite re-introduction in an area that is in the process of eliminating malaria. New outbreaks can be studied based on the current situation and new haplotypes can be easily identified.

## Conclusions

The *P. falciparum* diversity found in Ecuador could be a product of migration or the result of haplotypes circulating in the country in low proportions. The three genetic groups present in the north coast of Ecuador confirm the low transmission situation of the last endemic area of the coast of the country. Studies of the genetic characterization of *P. falciparum* in eliminating areas help determine the possible origin of parasites in order to create strategies to prevent the entrance of new lineages and achieve local elimination of malaria.

## Data Availability

All data generated or analysed during this study are included in this published article (and its additional files).

## Abbreviations

AMOVA: analysis of molecular variance
ART: artemisinin
BV_1_: genetic lineage BV1 variant
CQ: chloroquine
EV_1_: genetic lineage EV1 variant
Fst: pairwise fixation indices
He: heterozygosity
K: number of genetic groups
LD: linkage disequilibrium
MQ: mefloquine
PET-PCR: photo-induced electron transfer polymerase chain reaction
QN: quinine
RDT: rapid diagnostic tests
SP: sulfadoxine–pyrimethamine
